# Polymeric Patches Based on Chitosan/Green Clay Composites and Hazelnut Shell Extract as Bio-Sustainable Medication for Wounds

**DOI:** 10.3390/pharmaceutics15082057

**Published:** 2023-07-31

**Authors:** Carmen Laura Pérez Gutíerrez, Alessandro Di Michele, Cinzia Pagano, Debora Puglia, Francesca Luzi, Tommaso Beccari, Maria Rachele Ceccarini, Sara Primavilla, Andrea Valiani, Camilla Vicino, Maurizio Ricci, César Antonio Viseras Iborra, Luana Perioli

**Affiliations:** 1 Department of Pharmaceutical Sciences, University of Perugia, 06123 Perugia, Italy; carmenlaura.perezgutierrez@studenti.unipg.it (C.L.P.G.); tommaso.beccari@unipg.it (T.B.); mariarachele.ceccarini@unipg.it (M.R.C.); camivici@live.it (C.V.); maurizio.ricci@unipg.it (M.R.); luana.perioli@unipg.it (L.P.); 2 Department of Pharmacy and Pharmaceutical Technology, Faculty of Pharmacy, University of Granada, 18016 Granada, Spain; cviseras@ugr.es; 3 Department of Physics and Geology, University of Perugia, 06123 Perugia, Italy; alessandro.dimichele@unipg.it; 4 Department of Civil and Environmental Engineering, University of Perugia, UdR INSTM, 05100 Terni, Italy; debora.puglia@unipg.it; 5 Department of Materials, Environmental Sciences and Urban Planning (SIMAU), Polytechnic University of Marche, 60131 Ancona, Italy; f.luzi@staff.univpm.it; 6 Istituto Zooprofilattico Sperimentale dell’Umbria e delle Marche “Togo Rosati”, 06126 Perugia, Italy; s.primavilla@izsum.it (S.P.); a.valiani@izsum.it (A.V.)

**Keywords:** hazelnut shell extract, chitosan, green clay, bioadhesion, wounds, sustainability

## Abstract

Hazelnut shells, the main waste deriving from hazelnut processing, represent an interesting source of active molecules useful in pharmaceutics, although they have not yet been examined in depth. A hydrosoluble extract (hazelnut shell extract, HSE) was prepared by the maceration method using a hydroalcoholic solution and used as the active ingredient of patches (prepared by casting method) consisting of composites of highly deacetylated chitosan and green clay. In vitro studies showed that the formulation containing HSE is able to stimulate keratinocyte growth, which is useful for healing purposes, and to inhibit the growth of *S. aureus* (Log CFU/mL 0.95 vs. 8.85 of the control after 48 h); this bacterium is often responsible for wound infections and is difficult to treat by conventional antibiotics due to its antibiotic resistance. The produced patches showed suitable tensile properties that are necessary to withstand mechanical stress during both the removal from the packaging and application. The obtained results suggest that the developed patch could be a suitable product to treat wounds.

## 1. Introduction

The development of bio-sustainable formulations is a necessity due to the environmental impact of non-biodegradable pharmaceutical pollutants deriving both from the production processes and from expired/unused medicines that are not properly disposed of. Moreover, the problem of microplastics is growing rapidly and involves many excipients normally used in the pharmaceutical industry. In this regard, on 30 August 2022 the European Commission released a draft proposal intended to restrict intentionally added microplastics, and on 27 April 2023 the REACH committee expressed its positive vote to restrict the use of intentionally added microplastics, thereby representing an issue for the companies producing health products. Thus, the research of bio-sustainable materials is necessary for both excipients and active ingredients. From this perspective, the choice of natural sources can represent a valuable solution. Hazelnut (*Corylus avellana* L.) shells are one of the most abundant by-products deriving from hazelnut processing, and are mainly used for energy purposes [1]. It was demonstrated that they could represent a valuable source of active molecules useful in the health field. Extracts obtained from hazelnut by-products, rich in antioxidant molecules, could be used as active ingredients in nutraceutical products, dietary supplements, and pharmaceutical, dermatological, and cosmetic formulations [2]. Hazelnut shells are rich in polyphenols [3,4] responsible for many activities, such as antioxidant [4], antimicrobial [5], anti-inflammatory [6], immune-modulatory [7], and anti-cancer [8,9] properties. Despite the high potential for application in the health field, investigation of the development of formulations loaded with hazelnut shell extract is still limited, as demonstrated by the lack of papers in the literature. In our previous work a hydrosoluble hazelnut shell extract (HSE), characterized by high antioxidant activity mainly due to the high content of gallic acid, was successfully prepared by the maceration method [4]. It is noteworthy that gallic acid is a molecule that possesses high anti-oxidant capacity; this is useful in the protection from damage provoked by high levels of reactive oxygen species (ROS), which are responsible for oxidative stress [10]. It was found that oxidative stress plays a crucial role in delaying wound healing, as it is responsible for cell damage inducing a pro-inflammatory status. For this reason, the use of antioxidants in wounds treatment is considered a valuable strategy [11]. Many in vitro and in vivo studies demonstrated that gallic acid is able to stimulate growth of both keratinocytes and fibroblasts, suggesting its suitable use in wound healing stimulation [12,13,14].

Another important natural compound useful for stimulating wound healing is chitosan. Chitosan is a naturally occurring linear polysaccharide (isolated from the skeleton of crustaceans, insects, and cell walls of yeast and fungi), which is derived from chitin deacetylation and constituted by randomly distributed β-(l-4)-2-amino-2-Dglucosamine (deacetylated) and β-(l-4)-2-acetamido- 2 D glucosamine (acetylated) units. It was observed that chitosan participates in different stages in the process of wound healing. For example, it stimulates hemostasis, infiltration, and migration of neutrophils and macrophages; the expression of these growth factors is responsible for healing and re-epithelialization [15].

The combination of gallic acid and chitosan has been already tested and proven to be effective in the stimulation of wound healing. Park et al. developed thermosensitive gallic-acid-conjugated hexanoyl glycol chitosan that was able to induce both the expression of the growth factors involved in the wound healing process and the recruiting of fibroblasts [16]. Sun et al. developed biocompatible chitosan–copper–gallic acid nanocomposites that were able to enhance the healing rate of wounds infected by *S. aureus* due to their antimicrobial activity toward this specific strain [17]. Kaparekar et al. developed collagen–fibrin scaffolds based on chitosan nanoparticles loaded with gallic acid that were able to induce re-epithelialization and accelerate fibroblast cell migration and wound contraction [18]. In the literature, studies of the bioactive molecules (mainly polyphenols) contained in hazelnut shells can be found. However, the development of formulations intended for their administration is an unexplored field. This study investigated the possible application of a hazelnut shell extract, obtained by an eco-friendly method [4], in formulations intended for skin application for the treatment of diseases such as wounds. Bioadhesive patches are practical medications that can be used to cover wounds, providing at the same time protection from both mechanical and microbiological solicitation, as well as healing by means of proper active ingredients. With this in mind, HSE was formulated in bioadhesive, biocompatible, and biodegradable patches using chitosan as biopolymer and green clay as filler. The developed patches were studied in terms of mechanical properties and biological activity (wound healing and antimicrobial activity) in order to evaluate their possible application in wounds treatment.

## 2. Materials and Methods

BIO Hazelnuts (cultivar Tonda Gentile Romana) were obtained from Fattoria Lucciano Soc. Agr. s.s (Civita Castellana, Viterbo, Italy). Ethanol 96% (EtOH) was purchased from Sigma Aldrich (Milano, Italy). Chitosan FG90 (deacetylation degree 99.97%, MW 100 KDa, viscosity of 1% solution in 1% acetic acid 110 mPa s) was produced and characterized by Prof. Riccardo Muzzarelli, Department of Biochemistry, Biology and Genetics—Università Politecnica delle Marche-Ancona, Italy. Micronized green clay and glycerol were purchased from A.C.E.F. (Fiorenzuola D’Arda, Piacenza, Italy). Ultrapure water was obtained by a reverse osmosis process in a MilliQ system Millipore (Roma, Italy). Calcium chloride (CaCl_2_) was purchased from Carlo Erba (Milano, Italy). The simulated wound fluid (SWF) pH 6.5 was prepared by dissolving 8.30 g of NaCl and 0.28 g of CaCl_2_ in 1000 mL of ultrapure water [19].

Brain Heart Infusion (BHI) broth: deionized water 1000 mL, BHI (Biolife Italiana s.r.l., Milano, Italy) 37 g, pH: 7.4 ± 0.2, 25 °C.

Buffered Peptone Water (BPW): deionized water 1000 mL, Buffered Peptone Water (Biolife Italiana s.r.l., Milano, Italy) 20 g, pH: 7.0 ± 0.2, 25 °C.

Blood Agar with 5% Sheep Blood (BA): deionized water 1000 mL, Columbia Agar Base (Microbiol s.r.l., Cagliari, Italy) 44 g, Sheep Blood Defibrinated (Allevamento Blood di Fiastra Maddalena, Teramo, Italy) 50 mL, pH: 7.2 ± 0.2, 25 °C.

Human immortalized keratinocyte cell line (HaCaT) was purchased from I.Z.S.L.E.R. (Istituto Zooprofilattico Sperimentale della Lombardia e dell’Emilia Romagna) “Bruno Ubertini” (Brescia, Italy). Dulbecco’s Modified Eagle’s Essential Medium (DMEM), phosphate buffered saline (PBS), L-glutamine, trypsin, ethylenediaminetetraacetic acid disodium and tetrasodium salt (EDTA), Fetal Bovine Serum (FBS), and penicillin-streptomycin were purchased from Microtech S.r.l. (Pozzuoli, Napoli, Italy). 3-[4,5-dimethyl-2- thiazolyl]-2,5-diphenyl-2-tetrazolium bromide (MTT) and dimethyl sulfoxide (DMSO) were purchased from Merck (Darmstadt, Germany). Fixing solution and cell stain solution were purchased from Cell Biolabs, INC (San Diego, CA, USA).

### 2.1. Extract Preparation

Hazelnut shell extract (HSE) was prepared according to the method described in a previous work [4]. Briefly, 2 g of ground hazelnut shells (1 g having particle size of 710–1000 µm and 1 g having particle size of 500–710 µm) were kept overnight in 100 mL of EtOH 70%. Thereafter, the suspension was macerated at 45 °C for 300 min under magnetic stirring (1500 rpm) and then filtrated under vacuum by a Whatman 41 (Whatman GmbH, Dassel, Germany) cellulose filter membrane in order to recover the supernatant enriched with bioactive molecules. The solvent was then evaporated by a rotary evaporator (R-100, BUCHI, Cornaredo Italy) working at 35 °C. The concentrated product was dissolved in 5 mL of bidistilled water and freeze-dried (DRYWINNER, Heto, Gydevang, Denmark). The obtained hazelnut shell extract (hereinafter called HSE) was stored in CaCl_2_ until use.

### 2.2. Composite Patch Preparation

HSE was formulated in bioadhesive patches by the casting method [20] starting with gels prepared as follows. Green clay (0.5% *w/w*) was dispersed under magnetic stirring (600 rpm) in a water solution of lactic acid (1% *w/w*). The obtained suspension was then sonicated for 4 min at room temperature (R.T.) by the high-power ultrasonic (HPU) technique using an emitted power of 750 W and transmitted power of 200 W, frequency of 20 KHz, and amplitude of 50%, using a VCX750 horn-type ultrasonic probe (SONICS, Newtown, CT, USA). This passage was performed in order to obtain the clay exfoliation. Separately, chitosan gel was prepared using two chitosan percentages of 1.0 or 1.5% *w/v* (chitosan/green clay ratios = 2 and 3, respectively), in a lactic acid solution (1% *w/w*) in which the plasticizing agent glycerol was previously solubilized. The obtained chitosan gel was added to the exfoliated green clay suspension and sonicated by HPU for 4 min at R.T.

Loaded composites were prepared by dispersing HSE (0.065% *w/w*) under mechanical stirring (600 rpm) in the final gel and sonicated by HPU for 4 min. In order to remove the air incorporated during the preparation, all gels were degassed by an ARE-250 mixer (THINKY, Laguna Hills, CA, USA) working at 2000 rpm for 2 min at R.T. Then, 80 g of gel was cast in Teflon circular molds (diameter 14 cm) and left to dry in a ventilated oven for 24 h at 40 °C.

### 2.3. Morphology and Thickness

The patches’ morphology and thickness were evaluated by FE-SEM LEO 1525 ZEISS (Carl Zeiss Microscopy, Jena, Germany). The patches were positioned on conductive carbon adhesive tape and then metalized with chromium (8 nm) by sputtering.

### 2.4. Water-Holding Studies

The patches’ ability to absorb exudates was evaluated in vitro by calculating the hydration percentage (%) and matrix erosion (DS) using Equations (1) and (2), respectively:

(1) $$\mathrm{Hydration}\;\%=\frac{W2-W1}{W2}\times 100$$

(2) $$\mathrm{DS}\;\%=\frac{W1-W3}{W1}\times 100$$

A patch portion of 4 cm^2^ (2 cm × 2 cm) was weighed (W1), immersed in SWF (5 mL), and held at 32.0 ± 0.1 °C for established times (1, 2, 3, 4, 5, 6, 24, 48 h). After immersion, the samples were wiped using filter paper to remove the excess SWF and weighed (W2). After hydration, patches were dried at 60 °C for 24 h, maintained over CaCl_2_ (relative humidity R.H. 40%) for 48 h, and reweighed (W3) in order to calculate the DS% [20].

### 2.5. Ex Vivo Adhesion Studies

The patches’ ability to bind skin was evaluated ex vivo using samples (shoulder region) obtained from Large White pigs (weight ∼ 165–175 kg, supplied by Veterinary Service of ASL N. 1—Umbria, Città di Castello, Perugia, Italy). Skin samples were used for the assays within 12 h of pig death [21]. The patch was cut into portions of 2 cm × 2 cm and attached by cyanoacrylate glue to a support, which was then connected to a Didatronic dynamometer (Whatman GmbH, Dassel, Germany). A portion of porcine skin tissue was fixed by cyanoacrylate glue on the surface of a glass support placed in a thermostatic bath at 32.0 ± 0.5 °C. The free side of the skin was wetted using 50 μL of SWF and put in contact with the patch sample by applying a light force for 1 min. The force necessary for detachment of the patch from the skin was measured and is expressed as the average of three measurements (n = 3 ± SD).

### 2.6. FT-IR Analysis

Infrared (IR) spectra were registered by a Shimadzu IR Spirit QATR-S spectrometer. The spectra (4000–400 cm^−1^) of the samples were obtained against air as a background and a total of 100 scans were performed.

### 2.7. Mechanical Characterization

The tensile characterization was performed by a universal test machine (Lloyd Instruments LR30K). Patches were cut into rectangular samples of 100 mm × 10 mm (UNI ISO 527). The experiments were performed at 5 mm/min, a cell load of 50 N, and a useful length of 50 mm. The experiments were performed at R.T. and room humidity (R.H.). Values for maximum strength, deformation at break, and elastic modulus were registered. The reported results are an average of five measurements (n = 5). The samples were placed in desiccators containing a saturated MgCl_2_ solution for 1 week at R.T. until reaching constant weight before testing.

### 2.8. Cytotoxicity

The human immortalized keratinocyte cell line HaCaT was used as a model of the epidermidis. HaCaT was cultured according to standard procedures and maintained in an exponential growth phase in Dulbecco’s Modified Eagle’s medium (DMEM), supplemented with 10% heat-inactivated Fetal Bovine Serum (FBS), 2 mM of L-glutamine, and antibiotics (100 U/mL penicillin, 100 μg/mL streptomycin), as previously described [22]. Cell viability was tested by MTT assay; each experiment was performed in triplicate and cell viability is expressed as a percentage relative to that of the control cells set at 100% [23]. This experiment was performed first for HSE, then for the unloaded patches and the loaded one (Patch#1.5-HSE). In the first case, HSE was dissolved in DMEM complete medium at a concentration of 8 mg/mL and then vortexed. The obtained solution was used to prepare different dilutions with DMEM, then incubated with cells for 24 h. In the case of the patches, both unloaded and loaded (Patch#1.5-HSE), these were positioned on the cellulose membrane (Whatman 41, Whatman GmbH, Dassel, Germany) between the two chambers of a vertical Franz diffusion cell (USP <1724> PermeGear, Inc., Bethlehem, PA, USA, diameter 20 mm). The receptor chamber was filled with DMEM as a receptor medium (15 mL), thermostated at 37.0 °C ± 0.5, and magnetically stirred (600 rpm). The donor phase was filled with 2 mL of DMEM. All openings, including the donor top and receptor arm, were occluded by parafilm to prevent solvent evaporation. After 24 h, the acceptor medium was recovered and incubated with cells as it was (not diluted) and diluted with fresh DMEM using the ratios of 1:2, 1:4, and 1:8 (n = 3 ± SD). Data from three independent experiments were analyzed by ANOVA. GraphPad Prism version 9.2.0.332 was used (GraphPad software, San Diego, CA, USA) for MTT analysis. Data are expressed as mean ± S.D.

### 2.9. Wound Healing Assay

A CytoSelect™ Wound Healing Assay Kit (Cell Biolabs, Inc., San Diego, CA, USA) was used to investigate in vitro the effect on wound closure of HSE released from the patch. A 24-well tissue culture plate containing properly treated inserts was used. HaCaT cells for these experiments were seeded in DMEM complete medium at the final concentration of 3 × 10^5^ wells in a 500 μL final volume. After 24 h, inserts were removed from wells, leaving the wound field. Then, the medium was carefully aspirated and the wells washed twice with PBS 1X to remove dead cells and debris [24]. Patch#1.5-HSE (1 cm^2^) was incubated for 24 h in DMEM (10 mL); then, the supernatant was used for incubation with cells. Migration into the wound field was determined using manual fixing with a cell stain solution according to the manufacturer’s instructions. To analyze cell migration, a picture of each scratch was taken of the same area of cells at 6, 12, and 24 h after incubation. Representative images, focused on the center of the wound field, were taken. Three sets of experiments in duplicate were performed. At least 3 fields for each condition were taken, and the numbers of migrating cells into scratched fields were calculated. The influence of compounds on wound closure was compared to that of the untreated control treated with DMEM complete medium [25]. To measure the % closure, the migration cell surface area was determined for each experiment. Migration cell surface = total surface area (immediately after removing the insert) − cell-free area (white area in the photograph). The percent closure of the wound field was calculated for three different treatment times: 6, 12, and 24 h. Migration into the wound field was determined as previously described [20]. Three independent experiments were performed in duplicate.

### 2.10. Antimicrobial Activity Assay

The experiment was performed on *Staphylococcus aureus* WDCM 00034, revitalized on BHI broth and incubated at 37 °C for 24 h, following the procedure reported in a previous work [20]. Three different broth cultures were set up in BPW, with a bacterial concentration of 1 × 10^3^ CFU/mL. The composite patches (1 × 1 cm), loaded and unloaded, were added respectively to the first two broth cultures, while the third one, with untreated bacterial suspensions, was used as a control. The suspensions were incubated at 37 °C and the growth was evaluated after 4 h, 8 h, 24 h, and 48 h of incubation. In order to count the number of CFUs, a set of serial dilutions (from the initial solution to 10^−8^ CFU/mL) was taken from each sample and 100 µL was spread onto BA plates. After 24 h of incubation at 37 °C, the colonies were counted and the number of CFUs was calculated in accordance with the ISO 7218:2007 standard, using Equation (3):

(3) $$N=\frac{\sum C}{V\times 1.1\times d}$$

∑C is the sum of the colonies counted on the two dishes from two successive dilutions;
*V* is the volume of inoculum placed in each dish, in mL;
*d* is the dilution corresponding to the first dilution retained.

### 2.11. Statistical Analysis

Results of mechanical experiments were analyzed by analysis of variance (ANOVA), using the Statgraphics Plus 5.1. Program (Manugistics Corp. Rockville, MD, USA). To differentiate samples, Fisher’s least significant difference (LSD) was used at the 95% confidence level.

The statistical analysis of the cytotoxicity experiments was performed by the one-way ANOVA test and the significance thresholds were set as * *p* < 0.01, ** *p* < 0.001, and *** *p* < 0.0001. The nonparametric Mann–Whitney test or Kruskal–Wallis test with the post hoc Dunn test were used.

## 3. Results and Discussions

### 3.1. Patch Preparation and Characterization

The most common patches used for topical application bind skin by means of adhesives. This represents a problem, especially for the patient, as they are painful and can cause damage of the surrounding, newly formed tissue during removal. Moreover, from the environmental point of view, many excipients currently used in pharmaceutical/dermatological/cosmetic formulations could be subject to ECHA restrictions on intentionally added microplastics. A solution could be represented by the development of skin self-adhesive patches, which are removable by washing, and developed using both active ingredients and excipients from natural sources. With this objective in mind, chitosan, a natural bioadhesive polymer, was selected as the main component for patch development. Chitosan is FDA-approved for use in both food and pharmaceutical industries. It is an interesting excipient possessing activities that can be exploited. As a matter of fact, it has been demonstrated that chitosan, with a low molecular weight and high deacetylation degree, possesses antimicrobial activity [26]. In order to also exploit this aspect, modified chitosan FG90, having a molecular weight of 100 kDa and deacetylation degree, was chosen [27]. A previous study showed its activity against the Gram+ bacterium *S. pyogenes* and against the Gram− bacteria *P. aeruginosa*, *K. pneumoniae*, and *E. coli* [26]. These bacteria, which are often involved in wound infection, are included in the WHO priority pathogens list for R&D of new antibiotics as these strains are resistant to many conventional antibiotics [28]. Patches were obtained by the casting method as described in Section 2. Many prototypes were prepared in order to find the most suitable, starting with gels (H1–H5) having the composition reported in Table 1. Chitosan gel was prepared using a water solution of lactic acid (1% *w/w*) in order to obtain a biocompatible gel, as lactic acid is a physiological constituent of biological fluids. Firstly, the amount of FG90 was fixed to 1% w/w and four patches (F1–F4) were prepared. F1 was prepared without a plasticizing agent while the patches F2, F3, and F4 were prepared using different percentages of glycerol as a plasticizing agent. In all cases, the obtained patches did not show suitable properties in terms of elasticity or mechanical strength (breakage during removal from the mold), which was attributable to the low mechanical resistance of the selected polymer. For this reason, the addition of a filler as a reinforcing agent was considered useful and, in order to maintain the sustainability of the formulation, green clay was chosen. This material is already used in cosmetic products for skin use. It is an inorganic excipient composed of silica, alumina, or aluminum oxide; ferric oxide; titanium dioxide; calcium oxide; magnesium oxide; manganese oxide; sodium oxide; or potassium oxide. In addition, other elements, metals, or ions are also present in some types of green clay, such as zinc, copper, sulfur, carbon, nitrogen, phosphorus, and chlorine. Green clay mainly owes its typical color to the high content of trivalent iron (Fe^3+^) [29].

The literature data report that green clay possesses antibacterial activity and is active in wound care thanks to its adsorbing and healing properties. The mechanism of action in terms of antibacterial activity of this clay is not yet well known, while its activity in wound care is confirmed by its ability to absorb large quantities of liquids on its surface, which is a very important requirement in the case of exuding wounds [29]. The green clay used as a patch filler is a powder showing micrometric particles and having a mean diameter of 19.84 ± 1.34 µm. Chitosan/green clay composite was prepared by dispersing the green clay in lactic acid (1% *w/w*) water solution using the high-power ultrasonic technique (HPU) (R.T., 3 min). The obtained dispersion was then added to chitosan gel and sonicated by HPU again for 4 min at R.T. Finally, the composite was degassed before casting. The introduction of the filler improved patch flexibility; however, the resulting product was not easily manageable. Thus, it was considered useful to improve the polymer content to 1.5% w/w by maintaining the unmodified % of green clay and glycerol (0.5 and 5.0 *w/w* respectively, Table 1). The obtained patch composite F6 (hereinafter called Patch#1.5) was easily removable from the mold, and was flexible and resistant, and was thus further characterized.

### 3.2. Morphology and Thickness

**Figure 1:**
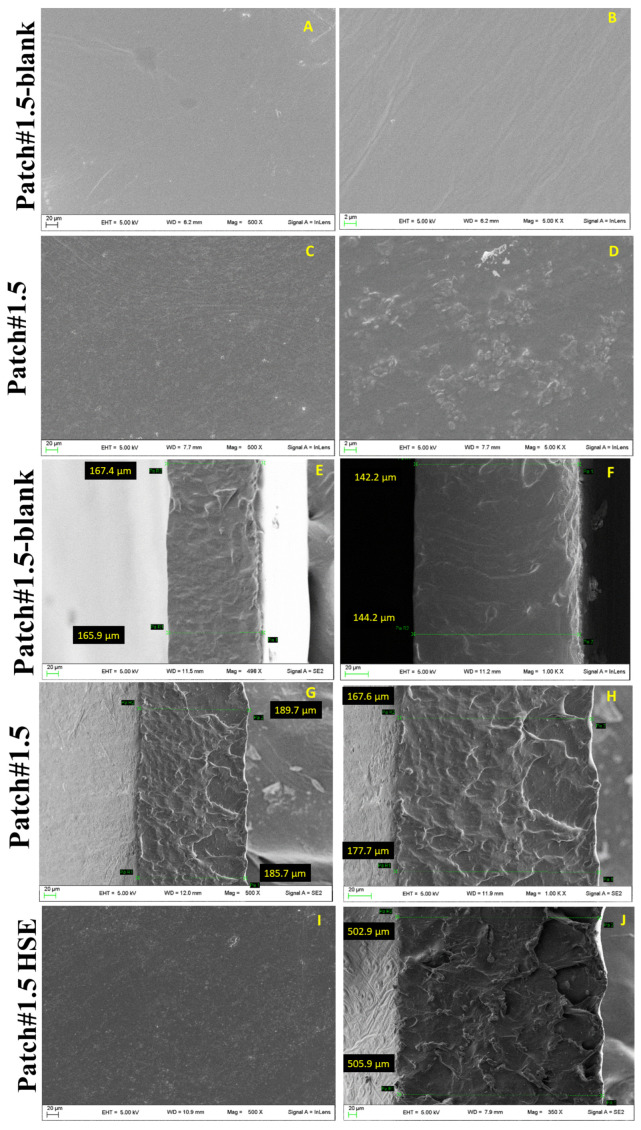
Micrographs of Patch#1.5-blank (**A**) magnification 500× and (**B**) 500 K ×, and Patch#1.5 magnification 500× (**C**) and 500 K × (**D**); thickness of Patch#1.5-blank (**E**,**F**) and Patch#1.5 (**G**,**H**); morphology of Patch#1.5-HSE (**I**) and thickness (**J**). Magnifications 500× and 100 K ×.

The morphology and thickness of Patch#1.5 were studied using scanning electron microscopy (SEM). For comparison, the same analysis was performed for Patch#1.5 prepared without green clay (named Patch#1.5-blank). Patch#1.5-blank shows a rather smooth and continuous surface (Figure 1A,B), while Patch#1.5 shows a wrinkled surface (Figure 1C,D) due to the presence of green clay. This is an important aspect that is beneficial to improving the superficial area in contact with skin, and thus the bioadhesion. The thickness of Patch#1.5-blank (Figure 1E,F) is 155 ± 13 µm and that of Patch#1.5 (Figure 1G,H) is 180 ± 10 µm. As expected, the introduction of green clay in the formulations is responsible for the increase in thickness.

### 3.3. Water-Holding Studies

The ability of Patch#1.5 to absorb exudates was evaluated in vitro by water-holding studies. The obtained results show that the hydration takes place rapidly after contact with simulated wound fluid (SWF). After 1 h, the amount of SWF absorbed was ~ 87% w/w, and this value was quite constant until the end of the experiment (with two slight decreases at 4 and 6 h), suggesting that the water uptake is fast.

This behavior could be explained by the presence of green clay, which is known for its excellent adsorbing properties. Furthermore, these results suggest that the formulation has an adequate swelling capacity, which is an important prerequisite both for SWF absorption and for obtaining an adequate release of the active ingredient at the application site.

**Figure 2:**
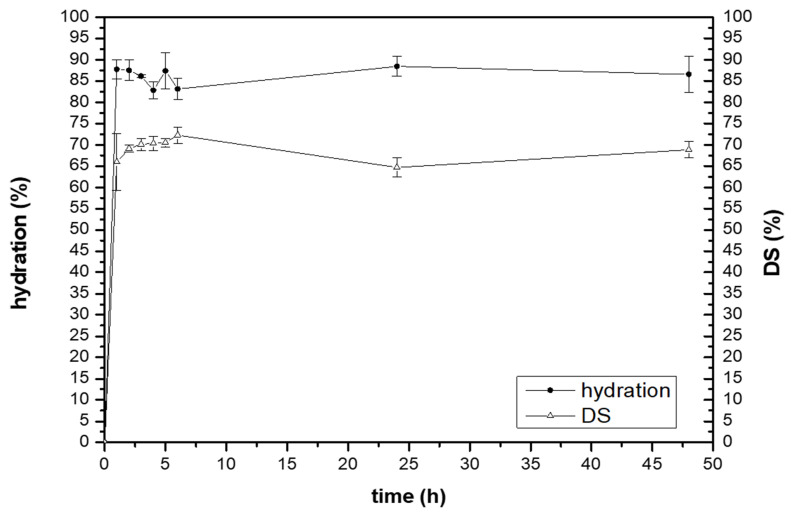
Hydration % and DS measured for Patch#1.5 at 32 °C in SWF.

Another important property that patches developed for wound treatment should possess is easy removal, which should keep the newly formed tissues intact and not cause pain to the patient. In order to investigate this aspect, the matrix erosion capacity or dissolution (DS) of Patch#1.5 was investigated. The obtained results (Figure 2) show that the DS value gradually increased from 1 h to 6 h, in which the value measured was ~ 72%, and then decreased slightly up to 48 h. This is likely due to chitosan erosion.

### 3.4. Ex Vivo Adhesion Studies

The bioadhesion ability of Patch#1.5 was evaluated using ex vivo studies [30]. The force measured for patch detachment from the pig skin was 0.55 ± 0.05 N. Considering the lipophilic nature of the stratum corneum, it could be hypothesized that the measured adhesion is the combination of hydrophobic interactions between the skin surface and the patch’s lipophilic groups. It has also been reported in the literature that chitosan interacts with the phospholipids of cell membranes, mainly through electrostatic interactions, including hydrogen bonding and hydrophobic forces, depending on the phospholipid packing density [31]. Considering the stratum corneum properties, it is possible to hypothesize that the establishment of hydrophobic interactions between the skin surface and the patch’s lipophilic groups is responsible for interfacial interactions. This allows rapid adhesion to the skin, while avoiding the use of adhesives, which are often toxic, painful, and uncomfortable.

### 3.5. Cytotoxic Activity

**Figure 3:**
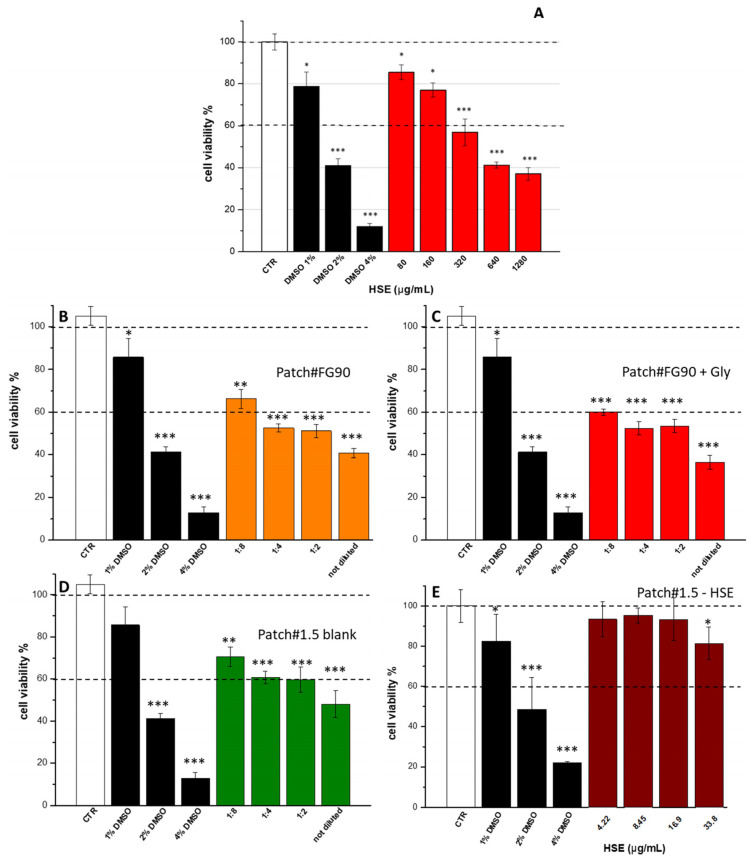
Viability of HaCaT cells incubated with (**A**) different concentrations of HSE. The control (CTR), in white, is represented by untreated cells in DMEM and set at 100%, whereas DMSO in black at three different percentages (1%, 2% and 4%) was used as positive control. The percentage of viable cells with respect to the control, in red, is reported as the mean ± SD of three independent experiments. Dotted lines indicate 100% and 60% cell viability. Supernatants were obtained from the incubation of the four different patches in DMEM: (**B**) Patch#FG90; (**C**) Patch#FG90 + Gly; (**D**) Patch#1.5-blank; and (**E**) Patch#1.5-HSE. * *p* < 0.01, ** *p* < 0.001, and *** *p* < 0.0001 vs. control one-way ANOVA test.

Hazelnut shell extract (HSE) was obtained according to a previous study [4] and characterized in order to evaluate its suitability for wound treatment. Before the loading in the selected formulation (Patch#1.5), HSE cytotoxicity was assayed on human keratinocytes (HaCaT) as a cell line representative of the stratum corneum. The resulting extract was safe in the concentration range of 80–160 µg/mL (Figure 3A), but become cytotoxic for concentrations of 320–1280 µg/mL. As Patch#1.5 was developed with the aim of using HSE to treat wounds, the same experiment was performed on the empty patch in order to evaluate the effect on cells of the raw materials used for its preparation. Thus, the experiment was performed on patches prepared using FG90 (Patch#FG90), FG90 + glycerol (Patch#FG90 + Gly), FG90 + glycerol + green clay (Patch#1.5), and Patch#1.5 + HSE (Figure 3).

**Figure 4:**
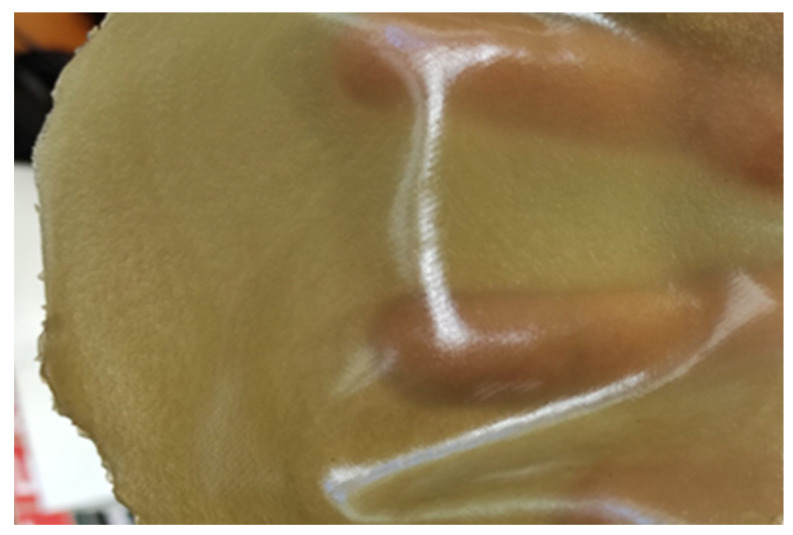
Picture of Patch#1.5-HSE.

The experiment was carried out as reported in Section 2. Each patch was placed on the top of a vertical Franz diffusion cell (in order to simulate the application on the skin surface), and both the acceptor and the donor chambers were filled with DMEM (HaCaT culture media) to simulate a release assay. After 24 h, the acceptor medium was recovered and incubated with HaCaT cells as it was (sample reported in Figure 3 as “not diluted”) and diluted with DMEM in the ratios of 1:8, 1:4, and 1.2 (DMEM recovered from the acceptor chamber/fresh DMEM *v/v*). The obtained results showed that, for Patch#FG90, the only dilution safe for cells (viability > 60%) was 1:8 (Figure 3B). The same situation was observed for Patch#FG90 + Gly (Figure 3C). Patch#1.5-blank was cytotoxic only for the not-diluted sample (Figure 3D). Patch#1.5-HSE was safe at all the concentrations tested (Figure 3E). In order to identify the reason for the limited safety, the pH of DMEM recovered from the acceptor medium after 24 h was measured. It was observed that this value varied slightly from 7.73 to 7.27 for Patch#FG90, whereas it was 7.30 for Patch#FG90+Gly and 7.63 for Patch#1.5-blank. The decrease in the pH value could be attributed to lactic acid present in patch compositions, which solubilizes. In the cases of Patch#FG90 and Patch#FG90+Gly, the pH decreased more than that of Patch#1.5, with a consequent increase in cytotoxicity. Thus, the obtained results suggest that Patch#1.5 is safe for cells. The decreased cytotoxicity is due to the capacity of green clay to bind lactic acid molecules by weak interactions, thereby avoiding their release in the medium. Thus, Patch#1.5 loaded with HSE (hereinafter called Patch#1.5-HSE, Figure 4), was prepared, starting with a gel having the following composition: 1.5% *w/w* CS FG 90, 0.5% *w/w* green clay, 5.0% *w/w* glycerol, 0.065% *w/w* HSE, and 92.935% *w/w* aqueous solution of lactic acid. Patch#1.5-HSE was prepared following the procedure described in Section 2. The amount was fixed to 0.065% *w/w* considering the cytotoxicity results for HSE (Figure 3A).

### 3.6. Loaded Patch Characterization

#### 3.6.1. Morphology and Thickness

The morphological analysis of Patch#1.5-HSE (Figure 1I) did not show considerable modifications compared to the empty patch (Patch#1.5, Figure 1C,D), while the thickness increased to 504 ± 2 µm (Figure 1J) as a consequence of the introduction of the extract.

#### 3.6.2. FT-IR Analysis

**Figure 5:**
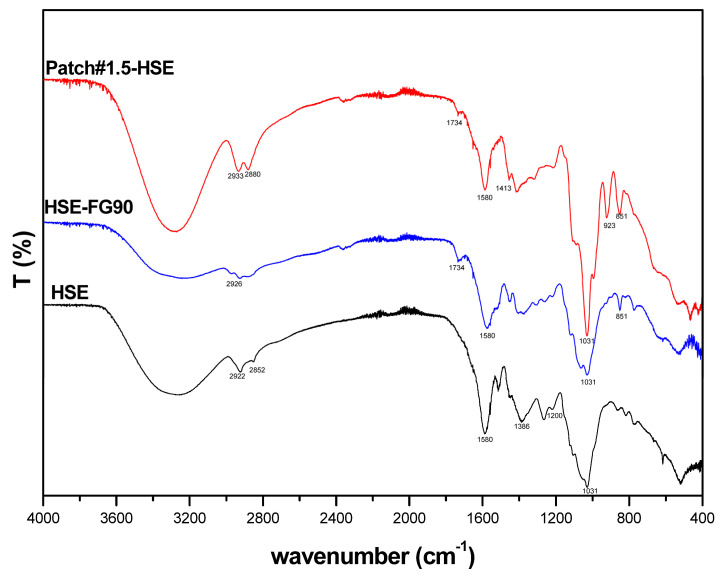
FT-IR spectra of HSE, HSE-FG90, and Patch#1.5-HSE.

FT-IR spectra were registered in order to evaluate possible interactions between HSE and the main components used for patch preparation, namely, FG90 and green clay (Figure 5). HSE is a mixture of phenolic compounds [4]; thus, the spectrum interpretation is complex due to the overlapping of signals coming from different molecules. A large band centered at 3300 cm^−1^ due to the symmetric and asymmetric stretching of hydroxyl groups found in phenolic compounds is visible. At 1580 cm^−1^, a peak due to the stretching vibration in the C–C ring is detectable. At ~1200 cm^−1^, a band due to the phenolic C–O stretching is detectable, and in the region between 1400 to 900 cm^−1^, bands attributable to C–H, C–O, and C–N bonds typical of phenolic compounds can be found [32]. The HSE-FG90 spectrum shows the same peaks as those found in the HSE spectrum, suggesting that no strong interactions occurred between these two compounds. For Patch#1.5-HSE, it is possible to detect typical peaks of the clay, such as at 1031 cm^−1^, attributable to the Si–O group, and at 923 cm^−1^, due to Al–OH–Al [33]. By observing the spectrum, it is possible to detect the peak at 1580 cm^−1^, which is also found in HSE. In the region between 1400 and 900 cm^−1^, there is the overlapping of signals coming from HSE, FG90, and green clay. Thus, it is difficult to assess the possible establishment of interactions. By observing the spectrum, it seems that no strong interactions occurred.

#### 3.6.3. Mechanical Properties

**Figure 6:**
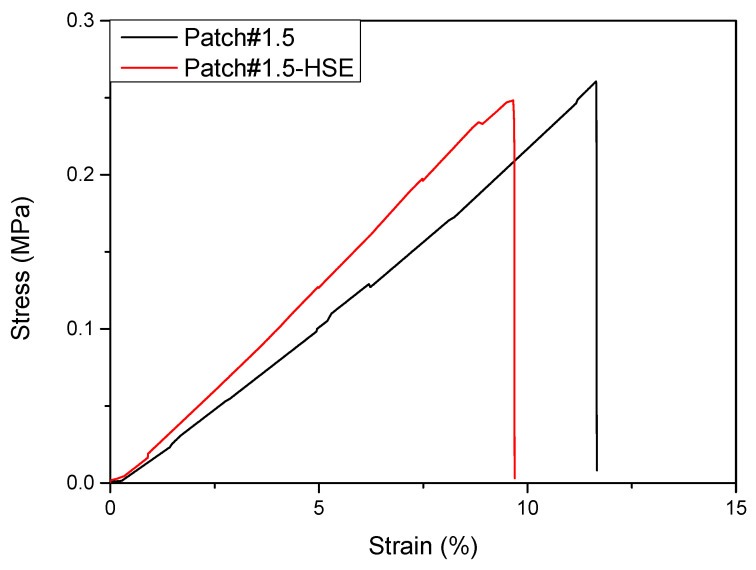
Tensile stress–strain curves for Patch#1.5 (black line) and Patch#1.5-HSE (red line).

The evaluation of the mechanical properties of both Patch#1.5 and Patch#1.5-HSE was also performed. The mechanical analysis of both patches is important as these systems were designed and developed to be used in direct contact with wounds. Additionally, for polymeric systems developed for wound healing, different characteristics are required for adhesion to different skin types and surfaces (smooth, wrinkled, or curved plate). For this aspect, detailed information about patches’ elastic response is necessary. It is necessary to clarify that the mechanical characteristics of hydrophilic and bio-based polymers are strongly influenced by relative humidity (RH), since the humidity acts as a plasticizer for the polymeric patch [34]. For each formulation, stress at break (σB), deformation at break (εB), and elastic modulus (E) were determined and are summarized in Table 2. The analysis of stress–strain curves of different developed systems (Figure 6) showed that Patch#1.5 is slightly more deformable than Patch#1.5-HSE. Similar values of stress at break were found by comparing Patch#1.5 with Patch#1.5-HSE. The presence of HSE increases the system stiffness (i.e., increases Young’s modulus). The enhanced mechanical properties observed for Patch#1.5-HSE can be related to the cross-linking role of HSE. This behavior is a result of stronger binding between HSE phenolic compounds and chitosan chains, according to the literature data. In fact, a similar effect was observed using phenolic extract obtained from banana peels and added to chitosan-based formulations [35]. Balasubramaniam et al., as an example, showed that the increase in terms of stiffness for chitosan/PVA plasticized by glycerol, in the presence of ferulic acid, could be explained by its conjugation to chitosan [36]. Rafieian and coworkers [37] showed that the addition of Aloe vera increased the modulus of the films with a parallel decrease in elongation at break. In general, the incorporation of phenolic extracts with chitosan is responsible for changes in mechanical resistance, due to the interaction between the extracts’ hydroxyl groups and chitosan amide groups [38].

#### 3.6.4. In Vitro Wound Healing Capacity

**Figure 7:**
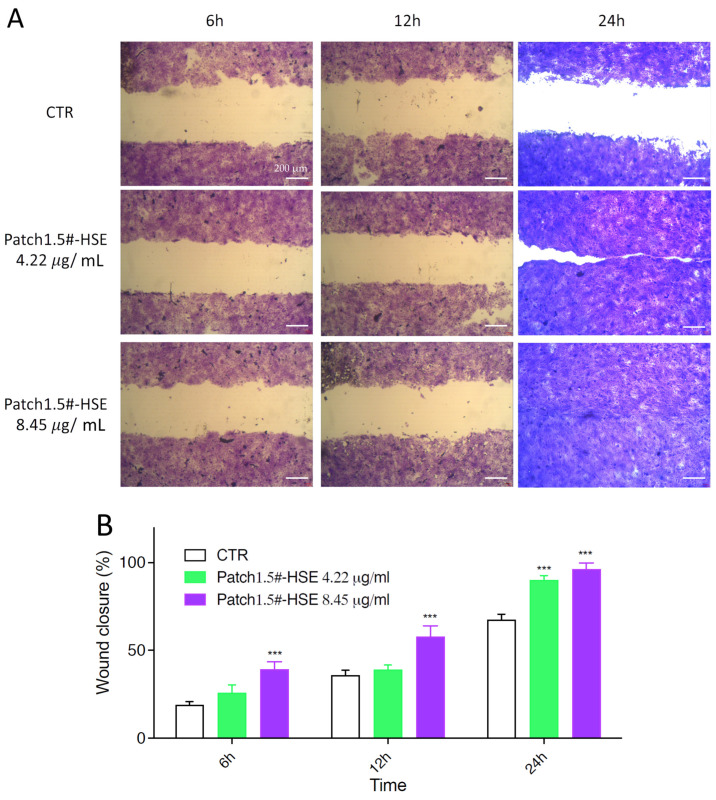
Wound healing was observed in three independent experiments after 6, 12, and 24 h of treatments. (**A**) Optical images in scratch test assay as representative images and (**B**) histogram plot together with ± SD are reported. Wound closure was calculated by manually tracing the cell-free area in captured images using the public domain software ImageJ (https://imagej.en.softonic.com/, NIH, Bethesda, MD, USA). Differences between untreated cells (in white) and cells treated with two different concentrations of Patch#1.5-HSE, namely, 4.22 (in green) and 8.45 μg/mL (in purple), were calculated using a one-way ANOVA test. *** *p* < 0.0001.

A further study was performed on the HaCaT cell line with the aim of evaluating the ability of HSE released from the loaded patch to stimulate cell growth, and thus the healing capacity of the amount released within 24 h (Figure 7). Based on MTT results, HSE was found to be safe for HaCaT cells at the lowest concentrations tested, namely, 0.08 and 0.16 mg/mL (80 and 160 µg/mL). A safe profile was obtained for Patch#1.5-HSE as the HSE concentrations produced were lower than the toxic ones. The best result in the scratch assay was obtained for an HSE concentration of 8.45 µg/mL after 24 h of treatment (Figure 7). In fact, compared to CTR, for which the wound field was still open (around 80%), a complete closure of the wound field (100%) was observed for the highest concentration tested (8.45 µg/mL). A concentration of 4.22 µg/mL also gave excellent results, as the wound field was almost closed after 24 h. After 3 and 6 h, no strong evidence was observed.

#### 3.6.5. Antimicrobial Activity

**Figure 8:**
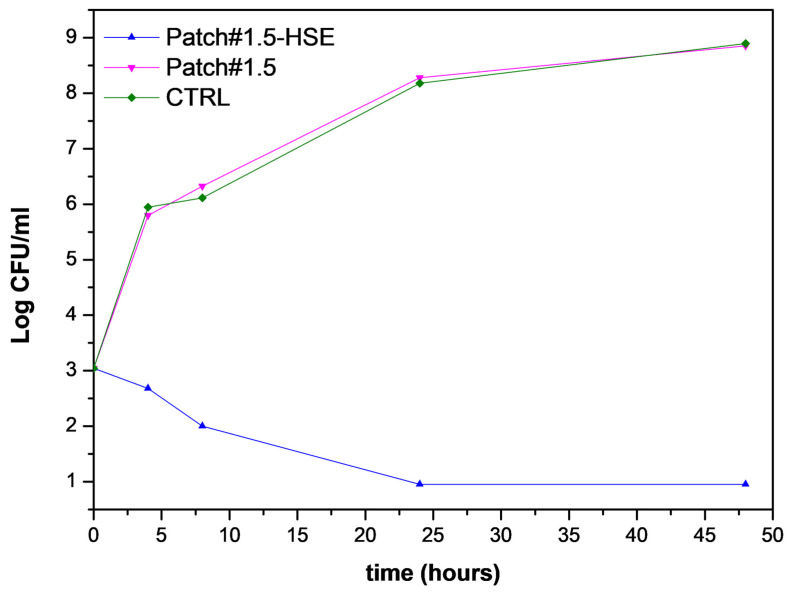
Growth curves of *S. aureus* in broth cultures treated with Patch#1.5-HSE compared to Patch#1.5 and CTRL samples.

Patch#1.5-HSE antibacterial activity was assayed because a previous study demonstrated HSE activity against *S. aureus* [4], which is a microorganism responsible for many skin infections. Moreover, it must be taken into account that *S. aureus* is reported in the WHO pathogens list of antibiotic-resistant pathogens [24]. Because of the increased resistance to conventional antibiotics, there is an urgent need for new antimicrobial agents that are able to treat the infections induced by this microorganism. In order to evaluate the performance of the final formulation, the experiment was performed on both the loaded patch (Patch#1.5-HSE) and the unloaded patch, which was used as the control (Patch#1.5). The obtained results (Figure 8) show that, in the bacterial suspension treated with Patch#1.5-HSE, no viable microorganisms were found after 24 h of incubation. In the same experimental conditions, in the CTRL and Patch#1.5 broth cultures, *S. aureus* gradually reached a final concentration of 8.8 Log CFU/mL. Considering that the amount of HSE in the patch assayed (1 × 1 cm^2^) was completely dissolved in 1 mL of bacterial suspension, the resulting active concentration was 337.96 µg/mL.

These results suggest that patches loaded with HSE are able to inhibit *S. aureus* growth and this activity is very important, as it can limit the onset of infections from the first hours after deposition on the wound.

## 4. Conclusions

A hydrosoluble hazelnut shell extract (HSE) was successfully formulated in bioadhesive patches, based on chitosan as a bio-polymer and green clay as a filler, intended for wound treatment. In vitro studies demonstrated that HSE released from the developed formulation is able to stimulate keratinocyte growth at very low concentrations (8.45 µg/mL). Moreover, the ability of HSE to suppress the growth of *S. aureus*, which is a bacterium notoriously involved in many wound infections, was demonstrated. This is of particular interest as HSE could be a valid alternative to the use of conventional antibiotics for which the antimicrobial resistance is a considerable problem. The results suggest that HSE could represent a valuable substitute to conventional drugs used in wound treatment and its use for other applications in health field is worthy of investigation.

## Figures and Tables

**Table 1:** Compositions of the starting gels used to prepare the patches. Patches’ aspects after solvent removal from the gel.

Gels	CS FG90 % (*w/w*)	Green Clay % (*w/w*)	Glycerol % (*w/w*)	Aqueous Solution of Lactic Acid % (*w/w*)	Final Patch Name	Aspect of the Obtained Patch
H1	1.0	-	-	99.0	F1	rigid
H2	1.0	-	10.0	89.0	F2	sticky
H3	1.0	-	5.0	94.0	F3	fragile
H4	1.0	-	2.0	97.0	F4	rigid
H5	1.0	0.5	5.0	98.5	F5	flexible but fragile
H6	1.5	0.5	5.0	93.0	F6	flexible and manageable

**Table 2:** Tensile parameters for Patch#1.5 and Patch#1.5-HSE.

Formulation	*σ_B_* (MPa)	*ε_B_* (%)	*E* (MPa)
Patch#1.5	0.29 ± 0.04 ^a^	11.63 ± 0.05 ^a^	1.95 ± 0.07 ^a^
Patch#1.5-HSE	0.32 ± 0.08 ^a^	11.54 ± 1.71 ^a^	2.03 ± 0.21 ^a^

(^a^) the same superscript within the same column indicates no significant differences among formulations (*p* < 0.05).

## Data Availability

Not applicable.
